# Using judgment bias test in pet and shelter dogs (*Canis familiaris*): Methodological and statistical caveats

**DOI:** 10.1371/journal.pone.0241344

**Published:** 2020-10-27

**Authors:** Carlotta Burani, Shanis Barnard, Deborah Wells, Annalisa Pelosi, Paola Valsecchi

**Affiliations:** 1 Dipartimento di Medicina e Chirurgia, Università degli Studi di Parma, Parma, Italia; 2 Dipartimento di Scienze Chimiche, della Vita e della Sostenibilità Ambientale, Università degli Studi di Parma, Parma, Italia; 3 Animal Behaviour Centre, School of Psychology, Queen’s University Belfast, Belfast, United Kingdom; University of Lincoln, UNITED KINGDOM

## Abstract

It is now widely agreed that a positive affective state is a crucial component of animal well-being. The judgment bias test represents a widespread tool used to assess animals’ optimistic/pessimistic attitude and to evaluate their emotional state and welfare. Judgment bias tests have been used several times with dogs (*Canis familiaris*), in most cases using a spatial test with a bowl placed in ambiguous positions located between a relatively positive trained location (P) which contains a baited bowl and a relatively negative trained location (N) which contains an empty bowl. The latency to approach the bowl in the ambiguous locations is an indicator of the dog’s expectation of a positive/negative outcome. However, results from such tests are often inconclusive. For the present study, the judgment bias test performance of 51 shelter dogs and 40 pet dogs was thoroughly analysed. A pattern emerged with shelter dogs behaving in a more pessimistic-like way than pet dogs. However, this difference between the two populations was detected only when analysing the raw latencies to reach the locations and not the more commonly applied adjusted score (i.e. average latency values). Furthermore, several methodological caveats were found. First of all, a non-negligible percentage of dogs did not pass the training phase, possibly due to the experimental paradigm not being fully suited for this species. Second, results showed a high intra-dog variability in response to the trained locations, i.e. the dogs’ responses were not consistent throughout the test, suggesting that animals may not have fully learned the association between locations and their outcomes. Third, dogs did not always behave differently towards adjacent locations, raising doubts about the animals’ ability to discriminate between locations. Finally, a potential influence of the researcher’s presence on dogs’ performance emerged from analyses. The implications of these findings and potential solutions are discussed.

## Introduction

It is now widely agreed that a positive affective state (comprising both the longer term background mood state and shorter-term discrete emotions [1]), and not only physical health, is a crucial component of animal well-being [1]. Animal welfare science and biomedical research have attempted to establish scientific and reliable measures of affective states in non-human animals, with the cognitive bias approach being one of the most commonly applied tests across species (for reviews see [2–5]; for critical analyses see [6, 7]; for a meta-analysis on how pharmacological manipulations influence judgment bias see [8]). Cognitive bias theories concern the influence of emotional states on cognitive functions (such as attention, memory and judgment) [3, 7]. These phenomena were initially studied in humans, pointing to a pre-attentive bias (selective attention towards threatening stimuli) in anxious people and a post-attentive bias (pessimistic appraisal of ambiguous stimuli) in depressed individuals [9].

Cognitive biases can be grouped into three main categories: attention biases, memory biases and judgment biases [2]. Judgment biases pertain to the optimistic/pessimistic interpretation of ambiguous stimuli and have been traditionally studied in humans using verbal tasks (e.g., the interpretation of ambiguous semantic sentences [10] and of lexical ambiguity such as homophones), which are typically linked to the individual’s mood (see for a review [11, 12]). However, in recent years, non-verbal tasks have been studied in humans in an effort to allow for comparisons with other animal species. Among others, Paul and colleagues [13] applied a computer-based spatial task in which subjects were required to decide whether an ambiguous stimulus (a cross located somewhere between a positive anchor point, i.e., 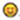, and a negative anchor point, i.e., 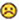) was nearer the positive or the negative anchor image. The authors found that subjects that presented higher “negative activation” and lower “positive activation” (i.e., a danger-oriented state and a state related to loss or absence of opportunity, respectively (measured using the PANAS scale, Positive and Negative Affect Schedule [14]), tended to interpret the cross as being nearer the negative anchor point. Anderson and colleagues [15] successfully applied a tone based task, developed in rats, to humans; participants were trained to correctly respond (left/right keypress on a serial response box) to a positive tone frequency in order to obtain rewards (money) and to a negative tone frequency in order to avoid punishment (an aversive sound clip). Subjects with higher self-reported measures of anxiety tended to respond to ambiguous tones (i.e., intermediate frequencies between the trained tones) in the same way they responded to the negative tone frequency [15].

Despite advancements in this field, caution is still required when using judgment bias tests (JBT) to assess the general mood of an individual. While studies using questionnaire and verbal tasks are generally suitable for this purpose, other studies, using non-verbal paradigms and measures such as response latency, may be only partially successful [16]. For example, IIgaya and colleagues [17] did not detect a relationship between self-reported mood and judgment bias using a visual task. Similarly, Schick and associates [16] found no significant correlation between mood and interpretation bias on a tone-based challenge.

Cognitive bias paradigms borrowed from human studies have been modified and adapted several times to measure the psychological welfare of many non-human species (e.g. rats [18], starlings [19], bees [20], sheep [21], macaques [22], pigs [23], horses [24], calves [25]), including the domestic dog, *Canis familiaris* [26–28]. Despite the large number of studies that have used JBT as the golden standard to evaluate the affective state of non-human animals, various questions remain to be answered; results are still not unequivocal and are not always in line with predictions to the point that some of those studies have led to null results or to opposite findings to those expected [7, 17, 29].

JBT results on dogs are as controversial as those involving other species. Some studies have found the expected relationship between emotions and judgment biases. For instance, Mendl and colleagues [27] applied a spatial JBT on dogs for the first time and found that sheltered animals scoring higher on separation-related behaviours showed a ‘pessimistic’ judgment of an ambiguous cue, suggesting a negative affective state. Titulaer and colleagues [30] employed physiological, behavioural and cognitive measures (spatial JBT) to evaluate potential differences between short-term and long-term sheltered dogs’ welfare. No cognitive bias differences were found between the two samples (in addition to no differences in physiological and behavioural measures); the authors concluded that other factors, e.g., frequency of social contacts, rather than the mere length of time spent in a shelter, can influence welfare state. More recently, Willen and colleagues [31] found that fearful sheltered dogs showed a more pessimistic expectation towards ambiguous stimuli than non-fearful kennelled dogs and that enrichment based on positive human interaction could increase positive expectancy in fearful animals. However, they also discovered that the same enrichment had the opposite effect on JBT results in non-fearful individuals.

Studies on dogs have not always reported the expected association between emotions and cognition. For example, Burman and colleagues [32], using a visual JBT, found that a brief positive experience (i.e., a food-based rewarding event) induced an unexpected pessimistic bias rather than an optimistic one; the authors explained that the previous food-based event may have reduced food motivation during the JBT and/or that the interruption of the positive experience could have elicited both negative emotions and pessimistic judgment bias. Müller et al. [33] used a spatial JBT and noticed that a brief absence of the owner was not sufficient to induce a pessimistic bias in pet dogs. More recently, Walker and colleagues [34] applied a spatial JBT to sheltered dogs and noticed that separation from their kennel-mate did not induce a pessimistic mood. More broadly, with regards the relationship between welfare state and judgment bias in sheltered dogs, Owczarczak-Garstecka and colleagues [35] found that the percentage of time spent asleep during the night was not predicted by dogs’ optimistic/pessimistic bias, while Harvey and colleagues [36] found that a more pessimistic judgment bias was unrelated to the amount of time dogs were awake, but inactive, in the home environment.

Some studies in this area, whilst reporting a significant association between emotions and cognitive biases in dogs, have reported methodological and statistical issues, including a small sample size that makes it hard to infer a general behavioural pattern [37, 38], the employment of a single ambiguous trial, the outcome of which could be influenced by momentary distraction [39, 40] and the use of a statistical approach that evaluates the average dog’s response instead of single trial responses, thereby reducing variability in the data [27, 31, 41]. Averaged measures are inevitably less accurate since, during the test, trials are repeated for each dog and for each type of cue and the number of repetitions is not consistent among cues (more trials for each trained cue, less trials for each ambiguous cue to minimize a potential learning effect).

Taken together, these equivocal results, sometimes even opposite to predictions, the occurrence of methodological caveats in the JBT paradigm and statistical concerns in data analysis suggest that a deeper investigation into the value of judgment bias tests as a measure of psychological welfare is necessary [42]. Given that the experimental paradigm has been modified several times (e.g., type of stimuli (spatial, auditory, visual), trained stimuli connotation (reward versus no reward, reward versus punishment), dependent variable (lever presses, latency to reach the stimuli, go/no go responses), number of ambiguous probes, learning criterion) and that statistical approaches employed to analyse the test output are not homogeneous, the reasons underlying these contradicting results are not easy to understand and further studies are required (for a review see [6, 7]).

Research on dogs is important as it allows us to investigate the basic emotional systems shared by social mammals [2] and offers the opportunity to compare different populations (i.e., pets, shelter, stray, laboratory, working dogs) that are often involved in welfare studies [43–49]. For the present research, a JBT with spatial stimuli was carried out with shelter-housed dogs, a population of animals ideally suited for studies on affective state. Entering a shelter is a stressful event for dogs [45], and the kennel environment can potentially induce negative emotions in more vulnerable individuals (due to stressors including noise, social deprivation, confinement). To exclude the possibility of our results being reflective of a population bias, data from a study on family pet dogs tested with the same JBT paradigm [26] were re-analysed alongside the data collected from the sheltered dog cohort.

The aim of this research was to evaluate the robustness and validity of the spatial JBT in dogs. To achieve this aim, the following points were thoroughly investigated:

- analysis of latencies to reach the five locations to ensure that dogs behaved differently towards them;
- analysis of the variability in latencies to reach the ambiguous locations to detect a potential learning effect over the trials;
- evaluation of dogs’ responses towards trained locations to check that subjects had correctly learned the association between the location and its outcomes.

Furthermore, a comparison between the two populations (sheltered vs. pet dogs) was made using both raw latencies to reach the bowl and an adjusted score of optimism/pessimism calculated using average latencies.

## Materials and methods

### Subjects

#### Shelter dogs

Fifty-one healthy sheltered dogs were selected for the study following two criteria: being in the shelter for at least 6 days and being aged between 1–11 years old. Thirty-five of these dogs passed the training phase (see later) and thus performed the JBT. These dogs varied in breed and were aged between 1–10 years old (average age: 4.19 ± s.d. 3.01 years). Twelve of the subjects were female (75% spayed) and 23 were male (39% neutered). Their average time spent in the shelter was 143 ± s.d. 301.38 days (min 6 days, max 1460 days).

Twenty-four dogs were recruited from the charity kennel “Benvardin Animal Rescue Kennels” (BARK), in Country Antrim, Northern Ireland (UK) and twenty-seven dogs enrolled were from the Italian Shelter “La cuccia e il nido” ruled in Calvatone (CR) by ANPANA (Associazione Nazionale Protezione Animali Natura Ambiente). In both shelters, dogs were either single or pair-housed. At BARK, dogs spent most of their day in outside enclosures (approximately 4x5 metres) and rested in smaller indoor pens (approximately 2x2 metres) during the night. In ANPANA shelter, dogs stayed in an indoor pen (approximately 3x2 metres) during the day and night and were released for exercise once or twice a day into outside enclosures (approximately 5x5 metres).

#### Pet dogs

Forty dogs were recruited among the students and staff of the School of Psychology, Queen’s University Belfast, and by word of mouth. Thirty-one of these dogs succeeded in the training phase (see later) and were therefore tested with ambiguous trials. These dogs, of various breeds, were between 1–10 years of age(average age: 4.55 ± s.d 2.57 years). Eighteen were females (78% spayed) and 13 were males (92% neutered). Owners provided consent for their pet to take part in the study and all of the animals were healthy. For a detailed report of dogs’ characteristics see Tables A and B in S1 Appendix.

### Judgment bias test

To avoid distractions, the JBT was carried out in a barren indoor area (approximately 6x6 m) within the two shelter facilities, and, for the pet dogs, in a testing room (5x5 m) at the Animal Behaviour Centre, Queen’s University Belfast. Data for the pet dogs were collected for another study [26] and raw data were re-analysed for this study.

The JBT protocol was the same as that adopted by others with dogs [27, 28]. In brief, it consisted of a spatial task with two trained cues (P-positive and N- Negative) and 3 ambiguous cues (NP-Near Positive, M-Middle, NN-Near Negative). Latency to reach the cue was evaluated: short latency indicates a potential anticipation of a positive outcome (food), i.e. an ‘optimistic’ judgment, whereas longer latency is reflective of a potential ‘pessimistic’ judgment. The test consisted of 2 phases: training and testing.

#### 1) Training

**Fig 1:**
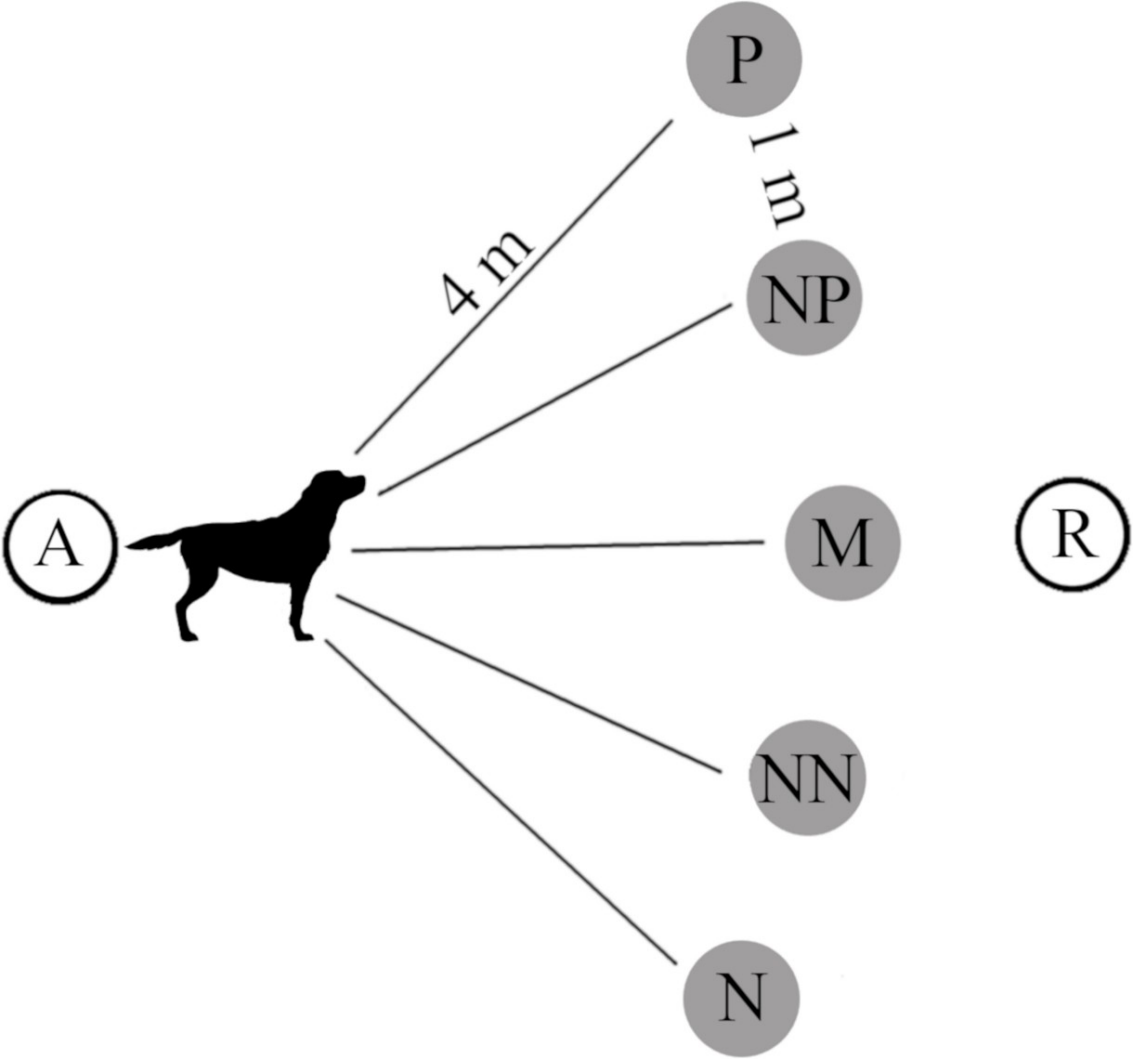
Experimental set-up. A (assistant), R (the researcher baiting/not baiting the bowl). Five bowl positions: P (Positive), NP (Near Positive), M (Middle), NN (Near Negative), N (Negative). Dog’s silhouette created using resources from Freepik.com.

Each dog was trained to discriminate between a positive location (P) in which a bowl baited with a piece of palatable food was placed on the ground, and a negative location (N) in which the bowl was always empty (see Fig 1 for locations of the bowl). The food used was a piece of sausage; to adapt the food intake to the dog’s size, we baited the bowl in positive trials with 1/2 slice of sausage for small/toy sized dogs, one slice of sausage (approximately weight = 3 gr) for medium sized dogs and two slices of sausage for large sized dogs.

The dog was presented with only one bowl per trial, placed in either the P or N position following a pseudo-random order, with no more than two trials of the same type performed consecutively. The location of the positive cue was balanced for the sample (i.e., for 50% of the dogs the positive cue was on the left-hand side of the room and for the remaining dogs was on the right-hand side). A researcher placed the baited/unbaited bowl on the ground in the P or N position while each dog was led on a leash to the starting position by an assistant (Fig 1).

The researcher pretended to bait the bowl to be placed in N, thereby reproducing the same movements and sound generated when the bowl was baited and then placed in P. The dog was allowed to watch the bowl baiting and positioning during the first 4 trials only (2P and 2N trials) to increase its motivation to check the bowl content. In all the other trials, the dog was led by the assistant behind a barrier during the bowl baiting in order to ensure that it could not observe what the researcher was doing; when the bowl was positioned, the researcher stood a couple of steps behind the M position, looking straight ahead and avoiding eye contact with the dog. The assistant then led the leashed dog to the starting position and released it after it was ensured that the animal had seen the bowl. Latency to reach the bowl (i.e., length of time from the point of release to the moment the dog’s nose was within 10 centimetres of the bowl) was measured using a stopwatch. The maximum latency to reach the bowl for each trial was set at 30 seconds; when this time elapsed, a latency of 30 seconds was recorded. The assistant then leashed the dog and led it to the starting position for the next trial.

Dogs had a minimum of 15 and a maximum of 40 trials to reach the learning criterion. This was set on the basis of other studies [26, 27, 32], so that for the preceding 3 positive trials and the preceding 3 negative trials, the longest latency to reach P was at least 0.5 seconds shorter than any of the latencies to reach N; this criterion was evaluated with each additional trial of training (rolling criteria). For each dog, the number of training trials required to reach the learning criterion was recorded.

#### 2) Testing

The testing phase took place immediately after the dog reached the learning criterion. Dogs were presented with an unbaited bowl placed in one of three ambiguous locations located between P and N, spaced equally along an arc, 4 metres from the starting position (see Fig 1). All probe locations were presented three times, separated by 4 standard training trials (i.e., P, N), following the order: M NP NN—NP NN M—NN M NP (each probe location was presented first, second or third in each block of three test trials). Overall, the testing phase included 41 consecutive trials.

At the end of the testing phase, an empty bowl was placed in the P position to make sure that the dogs were relying on spatial cues and not on the treat’s odour (42^nd^ trial, “false positive” bowl).

Overall, the judgment bias test (training + testing) lasted nearly one hour per dog.

### Statistical analyses

All analyses were performed using the software R, version 3.3.1. and packages DAAG (version 1.22), nlme (version 3.1–128), multcomp (version 1.4–6), MuMIn (version 1.40.4).

#### Analysis of the training phase

The effects of canine age (years), sex (male, female), reproductive status (neutered, intact)and position of positive bowl (left/right) on the number of training trials required to achieve the learning criterion was investigated using Spearman’s correlation tests for continuous variables and Mann-Whitney U tests or permutation tests for factor variables (a Levene test was used to check for homogeneity of variances). Data were analysed separately for the two populations of dogs (sheltered, pets). Furthermore, for the kennelled dogs only, the effect of length of stay (days) in the shelter and shelter location (Italy, N. Ireland) was explored using the same statistical analyses.

In addition, a Mann-Whitney U test was carried out to compare the number of training trials required to achieve the learning criterion between the sheltered and pet dogs.

#### Analysis of the testing phase

*Effect of the treat’s smell*. In order to ensure that dogs’ decision-making processes relied on bowl position and not on the odour of the treats, a Wilcoxon test for paired samples was employed to compare, for each dog, the median latency to reach the baited bowls placed in P during the test phase and the latency to reach the empty bowl placed in P on the last trial (“false positive” trial). This analysis was carried out separately for sheltered and pet dogs.

*Influence of dogs’ characteristics on latencies to reach the bowl*. As recommended by Gygax [42], a Linear Mixed-Effect model was applied (separately for sheltered and pet dogs), with untransformed latencies for each single trial (including the positive and negative cues used as anchor to estimate the slope across the different ambiguous cues) set as the dependent variable; a maximum-likelihood estimation was employed to account for designed imbalance in the data. This statistical approach has been criticized by Bateson and colleagues [50] who claimed that judgment bias regards only ambiguous stimuli and not the trained ones. Moreover, these authors considered ambiguous trails to be different from P/N ones because they are fewer (9 ambiguous trials vs. 32 P/N trials) and never reinforced. They therefore suggest not to pooling data from ambiguous and unambiguous trials and, rather, using data from the trained stimuli as a covariate in the analysis to account for differences in running speed between subjects. However, as pointed out by Gygax [42], the latencies to reach the trained locations are needed as anchors (dogs’ responses to trained locations are expected to be stable) to estimate a slope across the different ambiguous locations. Furthermore, proper random effects and a maximum-likelihood estimation can handle a designed imbalance in the data (i.e., different number of ambiguous and unambiguous trials) [51]. Using the dog’s identity as a random effect factor also takes into account the potential difference in running speed between subjects; in addition, using the average latency to reach the trained location as a covariate reduces the variability in this variable that was notable as regards to N location (see Results).

The Bayesian Information Criterion (BIC) was used to select the best combination of random effects. As expected, both in shelter and in pet dogs, the best models entailed that intercepts vary incorporating all the necessary hierarchical levels in the random effect (i.e., bowl location nested in dog identity). Model selection using BIC also showed that the model better fit the data if the bowl position variable was coded as a factor and not as a continuous variable; this could be ascribed to a deviation from the expected graded output (i.e., gradually higher latencies if the bowl was moved towards the negative position, see results for further explanations). Therefore, bowl position variable was coded as a factor in the analyses.

A backwards approach was applied to test the fixed effects of canine age, sex, reproductive status on the subjects’ latencies and, for the kennelled dogs only, the effects of length of stay in the shelter and shelter location (Italy, N. Ireland). These fixed effects were tested in interaction with bowl position and the interaction was removed from the model if not significant.

Since shelter location had no effect on latencies to reach the locations, the two kennelled populations were pooled for further analyses.

*Analysis of latencies to reach the five locations*. To assess whether the dogs behaved differently towards the bowl placed in the five positions, a Linear Mixed-Effects model (“lme” function) was created with bowl positions nested in dogs’ identity as a random effect, latency to reach the bowl as a dependent variable and bowl position as a fixed effect. To compare the behavioural responses of the two population, the dogs’ population (shelter/pet dogs) was included in the model in interaction with the bowl location variable.

Post-hoc tests with Bonferroni correction were used for pairwise comparisons between latencies to reach adjacent locations (P-NP, NP-M, M-NN, NN-N) and the two trained locations (P and N) separately for each population of animals. Furthermore, post-hoc tests with Bonferroni correction were used for pairwise comparison of latencies to reach the same location between the two populations.

*Variability in latencies to reach the ambiguous locations*: *Looking for a learning effect*. The analysis of latencies to reach the five locations (see results) showed that the variability in latencies was very high, suggesting a need for further analysis. With regard to ambiguous locations, variability in latencies could point to a learning effect, i.e., dogs after the first or the second trial may have learnt that the bowl placed in the ambiguous locations was empty; in that case, only the first trial would be a real ambiguous one and therefore only the first trial would be a reliable measure of optimism/pessimism. In order to explore for a potential learning effect, a Linear Mixed-Effects model was built for each ambiguous location (NP, M and NN), with dogs’ identity as a random effect, latency to reach the bowl as the dependent variable and the trial number (3 trials for each location, factor variable) in interaction with the dog’s population (shelter vs. pet dogs) as fixed effects; the interaction was removed from the model where not significant.

*Variability in latency to reach the trained locations*: *Intra and inter-dog components*. Consistency in dogs’ responses (estimated using the intra-dog variability in responses) was evaluated to check that subjects had correctly learned the association during the training. Based on the assumption that dogs should be confident in their responses towards trained locations whose outcomes they had previously learned, intra-dog variance in latencies to reach trained locations would be expected to be smaller than inter-dog variance. To evaluate the variability in dogs’ responses, two Linear Mixed-Effects models (one for P and one for N location) were built, with dogs’ identity as a random effect and latency to reach the bowl as the dependent variable. These models were used to split the total variance in the inter-dog (estimated variances between the random-effects terms in the linear Mixed-Effects model) and intra-dog (within-group error variance) components. Inter and intra-dog variances were compared for each trained location, separately for each population of animals.

*Dogs’ responses to the trained locations*. To determine how the subjects responded towards the trained stimuli (P and N), we evaluated the number of “go trials” and “no go trials”. A “go trial” is one in which the dog reaches the bowl within 30 s., a “no go trial” is a trial in which the dog does not reach the bowl within 30 s.

We calculated the percentage of “go trials” on the total number of negative/positive trials (number of N “go trials”/total number of N trials and number of P “go trials”/total number of P trials). A Pearson's Chi-squared test with Yates' continuity correction was also applied to evaluate if there was an association between the occurrences of “go/no go trials” and the dogs’ population (shelter vs. pet dogs), for both the P and N location. Standardised residuals and Cramér’s V effect size were evaluated.

*Optimism/pessimism adjusted scores*. Finally, to compare our results with previous studies, for each dog and each type of ambiguous location (NP, M and NN) an “adjusted score” of optimism/pessimism was calculated. We took into account each subject’s ‘baseline’ latencies to get to the trained stimuli (P and N), according to the following formula [27, 28]:

$$adjusted\;score=\frac{mean\;latency\;to\;probe\;location-mean\;latency\;to\;Positive\;location}{mean\;latency\;to\;Negative\;location-mean\;latency\;to\;Positive\;location}\;x\;100$$

An adjusted score near 0 means that the subject reacted to the probe location in a similar way to how it reacted to the positive location, i.e., it considered the ambiguous cue as potentially rewarding (optimistic bias); an adjusted score near 100 means that the subject reacted to the probe location in a similar way to how it reacted to the negative location, perceiving the ambiguous cue as potentially unrewarding (pessimistic bias).

We compared the optimism/pessimism adjusted score between sheltered and pet dogs using a Linear Mixed-Effects model with the dogs’ identity as a random effect, the adjusted score as the dependent variable and the bowl location in interaction with the dogs population variable as fixed effects; the interaction was removed from the model if not significant.

### Ethics statements

All applicable international, national, and/or institutional guidelines for the care and use of animals were followed. All procedures performed were approved by the School of Psychology Research Ethics Committee, Queen’s University Belfast (Ethical approval reference number No 90-2015-16). Special permission to use shelter dogs in such behavioural studies is not required in Italy (Decreto legislativo 4 marzo 2014, n. 26, Art. 2).

## Results

### Analysis of the training phase

None of the tested variables were significantly related to the minimum number of training trials required to reach the learning criterion, neither in sheltered, nor in pet dogs (see Table 1 for detailed results).

**Table 1:** Influence of dogs’ characteristics on the minimum number of training trials required to reach the learning criterion.

Tested variable	Population	Test statistic	P-value
Age	Sheltered dogs	Spearman’s correlation rho = -0.21	p = 0.23
	Pet dogs	Spearman’s correlation rho = -027	p = 0.15
Sex	Sheltered dogs	Permutation test with 50000 simulations	p = 0.17
	Pet dogs	Mann-Whitney U test W = 77	p = 0.11
Reproductive status	Sheltered dogs	Mann-Whitney U test W = 157	p = 0.91
	Pet dogs	Mann-Whitney U test W = 93.5	p = 0.13
Positive bowl location	Sheltered dogs	Mann-Whitney U test W = 174	p = 0.50
	Pet dogs	Mann-Whitney U test W = 128.5	p = 0.75
Length of stay in the shelter (days)	Sheltered dogs	Spearman’s correlation rho = -0.21	p = 0.23
Shelter location (Italy vs. N. Ireland)	Sheltered dogs	Mann-Whitney U test W = 147	p = 0.86

The minimum number of training trials required to reach the learning criterion did not differ significantly between sheltered and pet dogs (mean±sd 20.14±6.33 for sheltered dogs, 23.97±8.87 for pet dogs, Mann-Whitney U test W = 402.5: p = 0.07).

### Analysis of the testing phase

#### Effect of the treat’s smell

Wilcoxon tests for paired samples showed no statistically significant difference between median latencies to reach the baited bowls located in P and the latency to reach the empty bowl located in P in the last trial, either in sheltered dogs (z-value = 0.42, p = 0.39) or pets (z-value = 0.46, p = 0.20), confirming that the dogs’ decision-making processes relied on bowl location and not on the treat’s smell.

#### Influence of dogs’ characteristics on latencies to reach the bowl

Linear Mixed-Effects models did not reveal any significant effect of age, sex, reproductive status, length of stay (days) in the shelter or shelter location on latencies to reach the various bowl locations (see Table 2 for detailed statistical results).

**Table 2:** Analysis of the influence of dogs’ characteristics on latencies to reach the bowl.

Dogs’ characteristic	Population	F-value	df	P-value
Age	Sheltered dogs	0.5533	29	p = 0.46
	Pet dogs	0.35980	27	p = 0.55
Sex	Sheltered dogs	3.0975	29	p = 0.09
	Pet dogs	0.01773	27	p = 0.90
Reproductive status	Sheltered dogs	0.2656	29	p = 0.61
	Pet dogs	0.00157	27	p = 0.97
Bowl location * Age	Sheltered dogs	1.6450	116	p = 0.17
	Pet dogs	0.15006	108	p = 0.96
Bowl location * Sex	Sheltered dogs	0.8575	116	p = 0.49
	Pet dogs	0.55721	108	p = 0.69
Bowl location * Reproductive status	Sheltered dogs	0.1479	116	p = 0.96
	Pet dogs	0.36174	108	p = 0.84
Length of stay (days)	Sheltered dogs	0.0576	29	p = 0.81
Shelter location (Italy vs. N. Ireland)	Sheltered dogs	3.5648	29	p = 0.07
Bowl location * Length of stay (days)	Sheltered dogs	0.3587	116	p = 0.84
Bowl location * Shelter location	Sheltered dogs	0.4476	116	p = 0.77

* indicates the interaction between variables.

#### Analysis of latencies to reach the five locations

There was a significant interaction between bowl location and dogs’ population (shelter/pet dogs) on the animals’ latencies to reach the bowl (F-value = 6.7681, df = 256, p-value < .0001).Post-hoc contrasts analysis revealed that sheltered dogs behaved differently towards adjacent bowl positions, except for bowls located in P and NP (see Table 3). In contrast, pet dogs did not behave differently towards adjacent bowl positions, except for bowls located in the NN and N locations (see Table 3). Both sheltered and pet dogs successfully distinguished between bowls located at P and N (see Table 3).

**Table 3:** Pairwise comparisons between latencies to reach trained locations and adjacent ones.

Pairwise comparison	Population	Estimate	P-value
P–N	Sheltered dogs	-18.80	p <0.0001 ***
	Pet dogs	-13.65	p <0.0001 ***
P–NP	Sheltered dogs	0.43	p = 1.00
	Pet dogs	-0.13	p = 1.00
NP–M	Sheltered dogs	-4.37	p = 0.007 **
	Pet dogs	-2.41	p = 1.00
M–NN	Sheltered dogs	-5.47	p = 0.0002 ***
	Pet dogs	-2.86	p = 0.47
NN–N	Sheltered dogs	-14.42	p <0.0001 ***
	Pet dogs	-8.25	p <0.0001 ***

Simultaneous tests for general linear hypotheses, Multiple comparisons of means. P = Positive location, NP = Near Positive location, M = Middle location, NN = Near Negative location, N = Negative location. Significance codes: 0 ‘***’ 0.001 ‘**’ 0.01 ‘*’ 0.05, adjusted p values reported, Bonferroni method.

**Fig 2:**
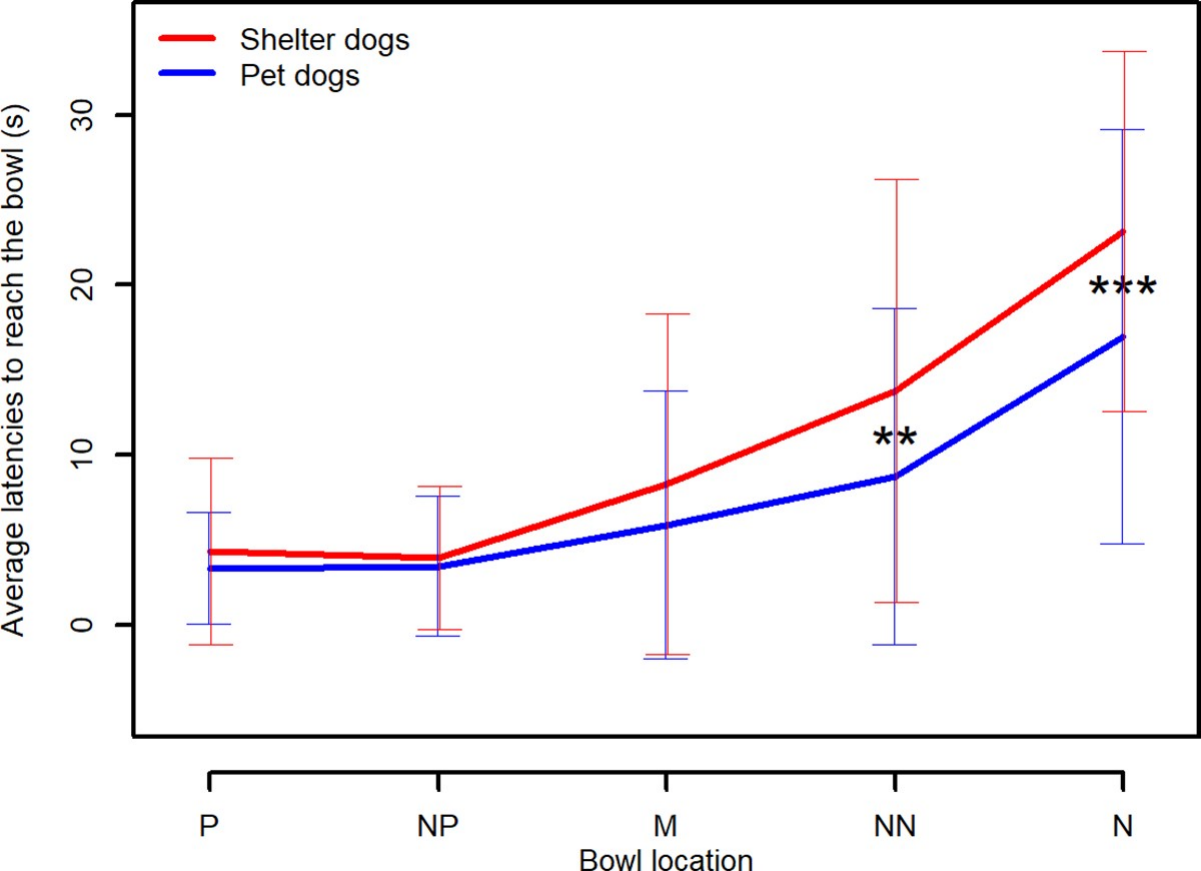
Average latencies to reach the five locations ± standard deviations, for sheltered and pet dogs. Positive (P), Near Positive (NP), Middle (M), Near Negative (NN) and Negative (N) location. * shows a significant difference (significance codes: 0 ‘***’ 0.001 ‘**’0.01).

Post-hoc contrasts analysis revealed that latencies to reach P, NP and M locations were not significantly different between sheltered and pet dogs, but a significant difference was found in the animals’ latencies to reach the NN and N locations (latencies were higher in shelter dogs, see Fig 2 for a graphic depiction and Table 4 for detailed statistical results).

**Table 4:** Pairwise comparisons between pet and sheltered dogs’ latencies to reach the five locations.

Location	Estimate	P-value
P	-1.0198	p = 1.00
NP	-0.4603	p = 1.00
M	-2.4237	p = 1.00
NN	-5.0333	p = 0.008 **
N	-6.1674	p <0.0001 ***

Simultaneous tests for general linear hypotheses, Multiple comparisons of means. P = Positive location, NP = Near Positive location, M = Middle location, NN = Near Negative location, N = Negative location. Significance codes: 0 ‘***’ 0.001 ‘**’ 0.01 ‘*’ 0.05, adjusted p values reported, Bonferroni method.

**Fig 3:**
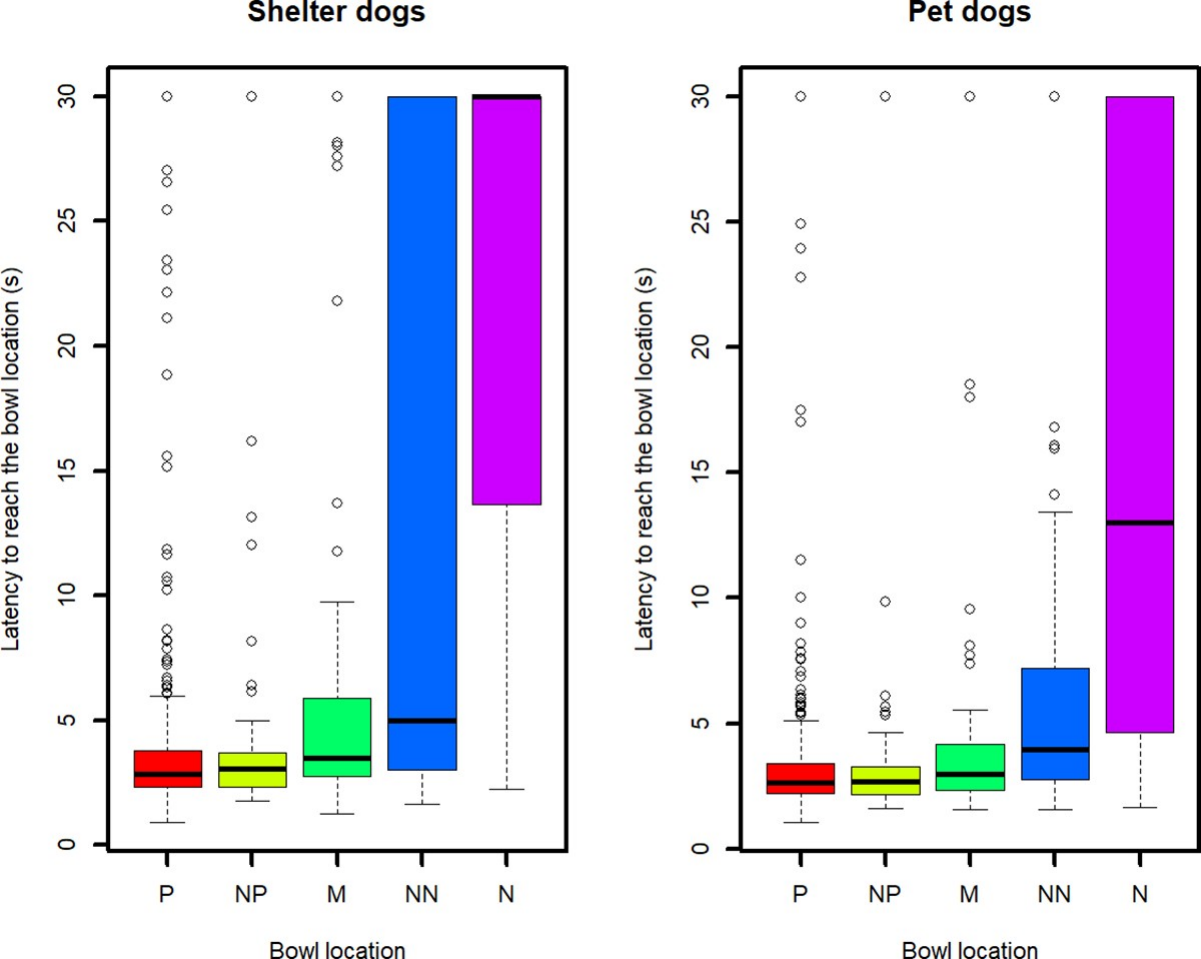
Boxplots of latencies to reach the five locations. Positive (P), Near Positive (NP), Middle (M), Near Negative (NN) and Negative (N) location. The graph shows medians (bar within the box), upper and lower quartiles (borders of box), lowest and highest cases within 1.5 times the IQR (bottom and top whiskers) and outliers (circles).

Despite many studies on JBT using mean values to graphically show an increasing general trend in latencies from P to N location [18, 27, 30, 32, 34], we used median values as these are more reliable and robust measures than means in cases of non normally distributed variables and extreme values (such as a 30 s latency recorded for a No-go response whereas the majority of trials presented a quick Go response). The general trend in median latencies to reach the five locations is graphically shown in Fig 3. It is worth noticing that the median latency to reach M, expected to be the most ambiguous location, is extremely short (3,48 s for shelter dogs, 2,96 s for pet dogs), almost equivalent to the median latency to reach P (2,84 s for shelter dogs, 2,66 s for pet dogs), suggesting a potential optimistic bias (see Discussion for an extensive explanation). Furthermore, Fig 3 shows that the variability in latencies is very high, suggesting the need for more in-depth analysis (see later).

#### Variability in latencies to reach the ambiguous locations: Looking for a learning effect

**Fig 4:**
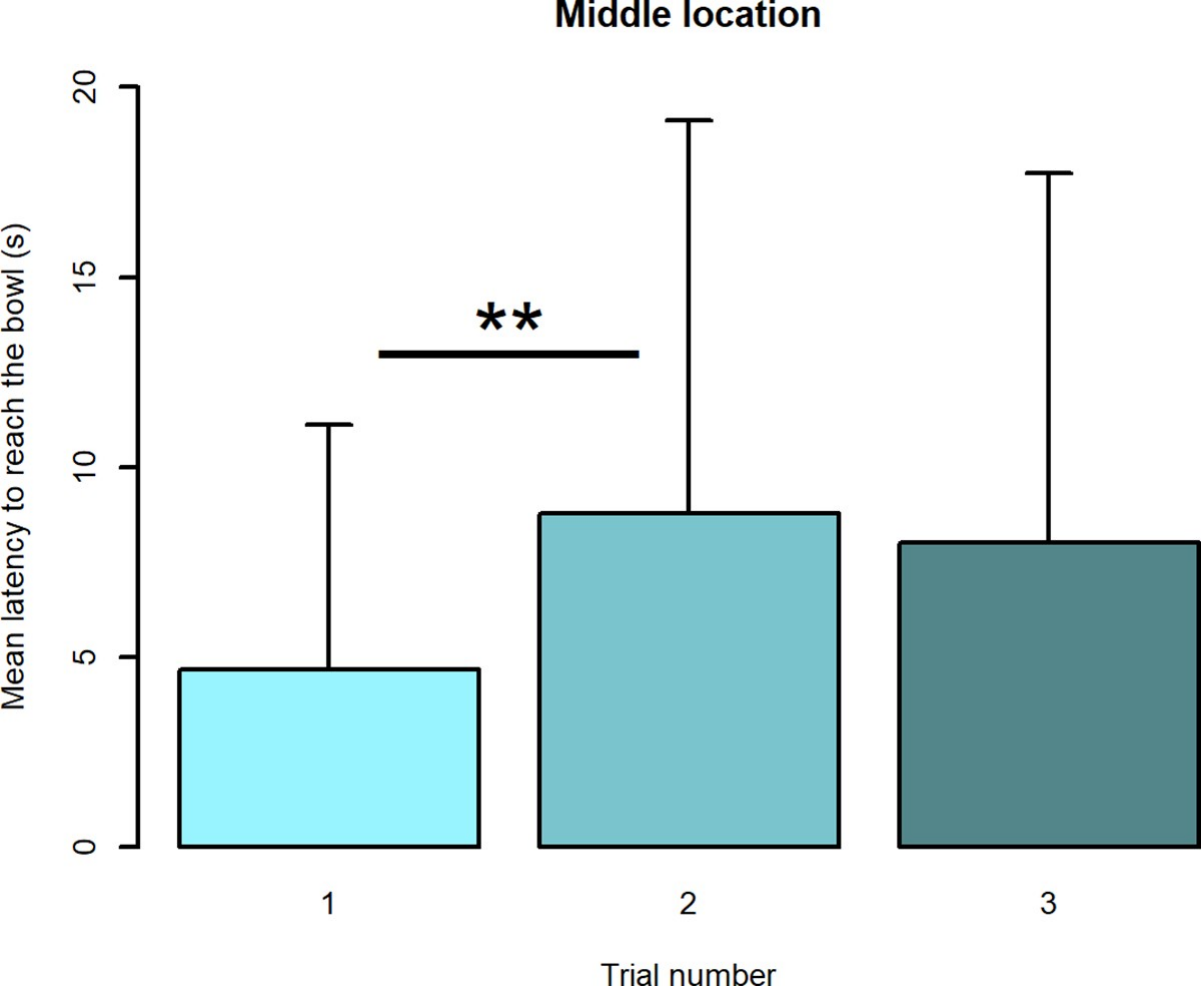
Barplot of latencies to reach the M location during the three trials. Means + sd are shown. ** shows a significant difference, p < 0.01.

Trial number was not found to have a significant effect on dogs’ latencies to reach the bowl placed at the NP location. However, trial number had a significant effect on both the sheltered and pet dogs’ latencies to reach the bowl positioned at M (df = 130, F-value = 5.17740, p-value = 0.007). Post-hoc contrasts analysis revealed that latencies to reach M were significantly higher in trial 2 than in trial 1 (estimate = -4.109848, df = 130, p-value = 0.003); latencies did not differ significantly, however, between trial 2 and trial 3 (see Fig 4).

**Fig 5:**
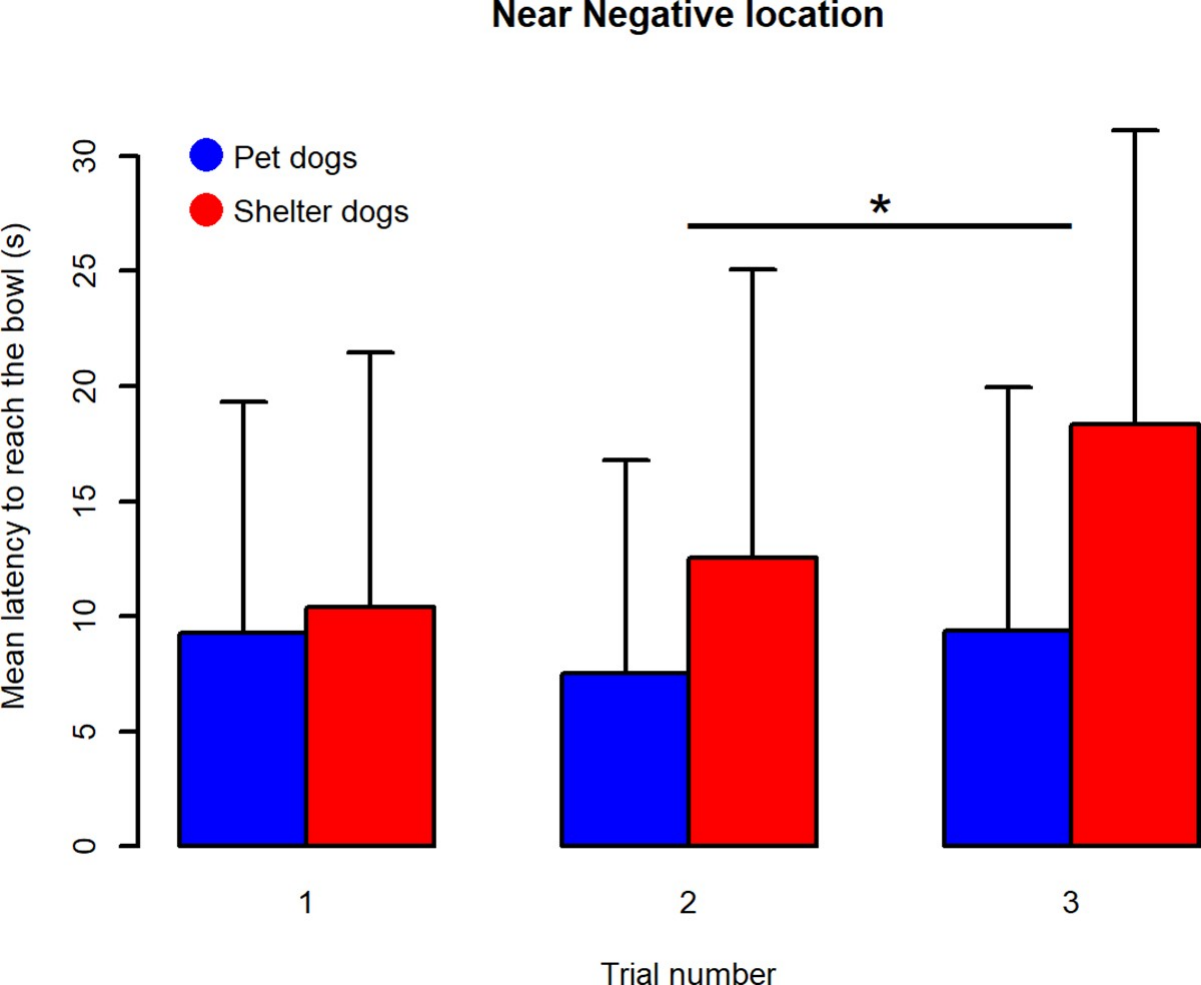
Barplot of latencies to reach the NN location during the three trials. Means + sd are shown. * shows a significant difference, p < 0.05.

Dog population was found to have a significant effect on animals’ latencies to reach the bowl positioned at NN, with higher latencies in sheltered dogs than in pets (estimate = 5.033266, df = 64, p-value = 0.01), according to the previous analysis (see analysis of latencies to reach the five locations). Latencies to reach the bowl in this position were also significantly related to trial number (df = 130, F-value = 3.68575, p-value = 0.03). Post-hoc contrasts analysis revealed that latencies to reach NN were not statistically different between trial 1 and trial 2, but were significantly higher in trial 3 than in trial 2 (estimate = 3.942879, df = 130, p-value 0.03, see Fig 5). The interaction trial * dog’s population was not significant.

#### Variability in latency to reach the trained locations: Intra and inter-dog components

**Fig 6:**
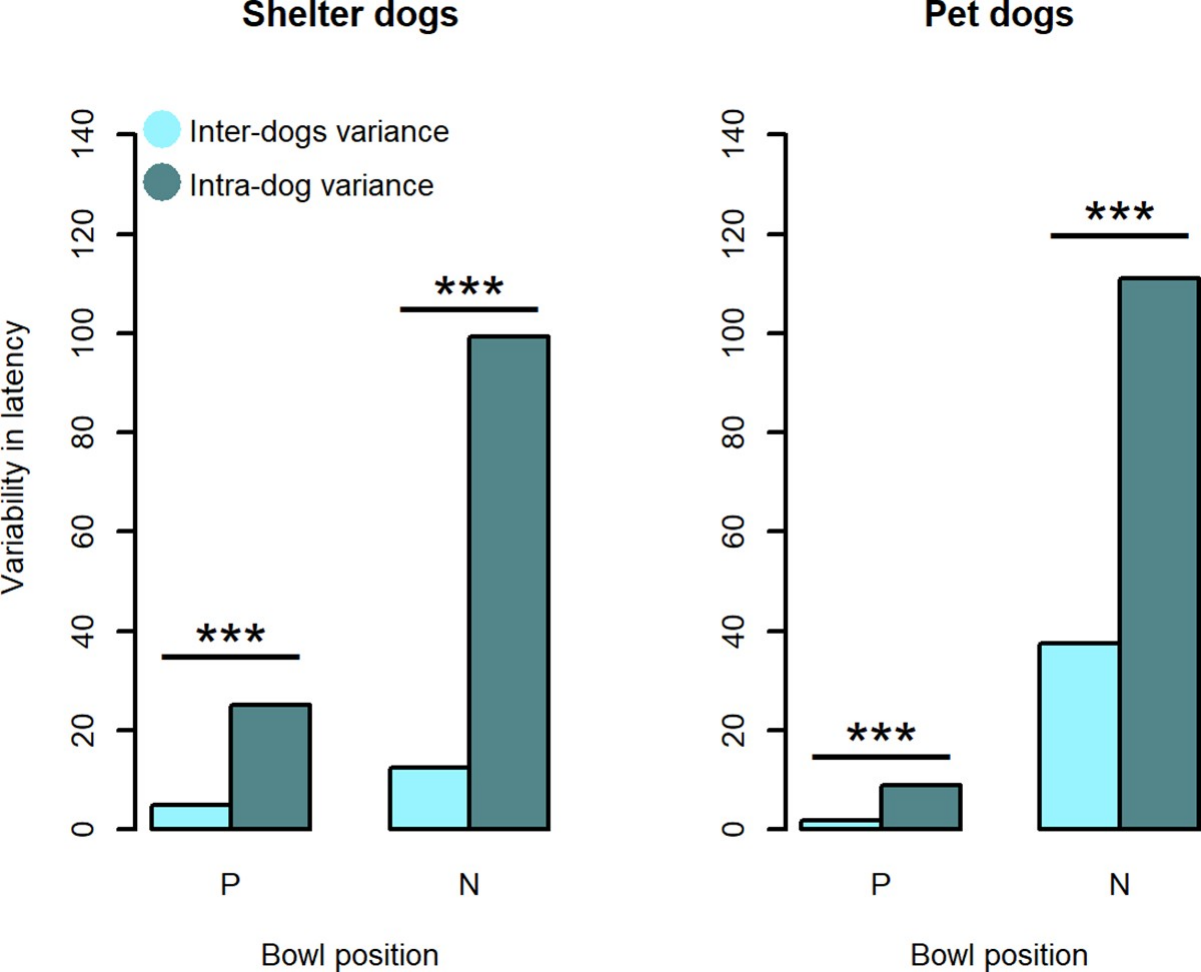
Inter and intra-dog variability in latency to reach the trained locations. Inter-dog (lighter bars) and intra-dog (darker bars) variance of latencies to reach the trained locations: Positive (P) and Negative (N). *** means p-value < 0.001.

We compared intra-dog and inter-dog variability in latencies to reach the bowl placed in the trained locations. Contrary to expectations, intra-dog variability was significantly higher than inter-dog variability, both for P location (in shelter dogs p-value < 0.0001, in pet dogs p-value < 0.0001) and N location (in sheltered dogs p-value < 0.0001, in pet dogs p-value < 0.001). Results are shown in Fig 6.

#### Dogs’ responses to the trained locations

During the testing phase, the percentage of ‘go trials’ for the P location was 96,79% for sheltered dogs and 99.19% for pet dogs (average latencies in ‘go trials’±sd were 3.50±2.93 s and 3.12±2.21 s for shelter and pet dogs respectively). The percentage of ‘go trials’ for the bowl positioned at N was 33.57% for sheltered dogs and 56.05% for pet dogs (average latencies in ‘go trials’±sd were 9.60±7.54 s and 6.78±5.30 s for sheltered and pet dogs respectively).

**Fig 7:**
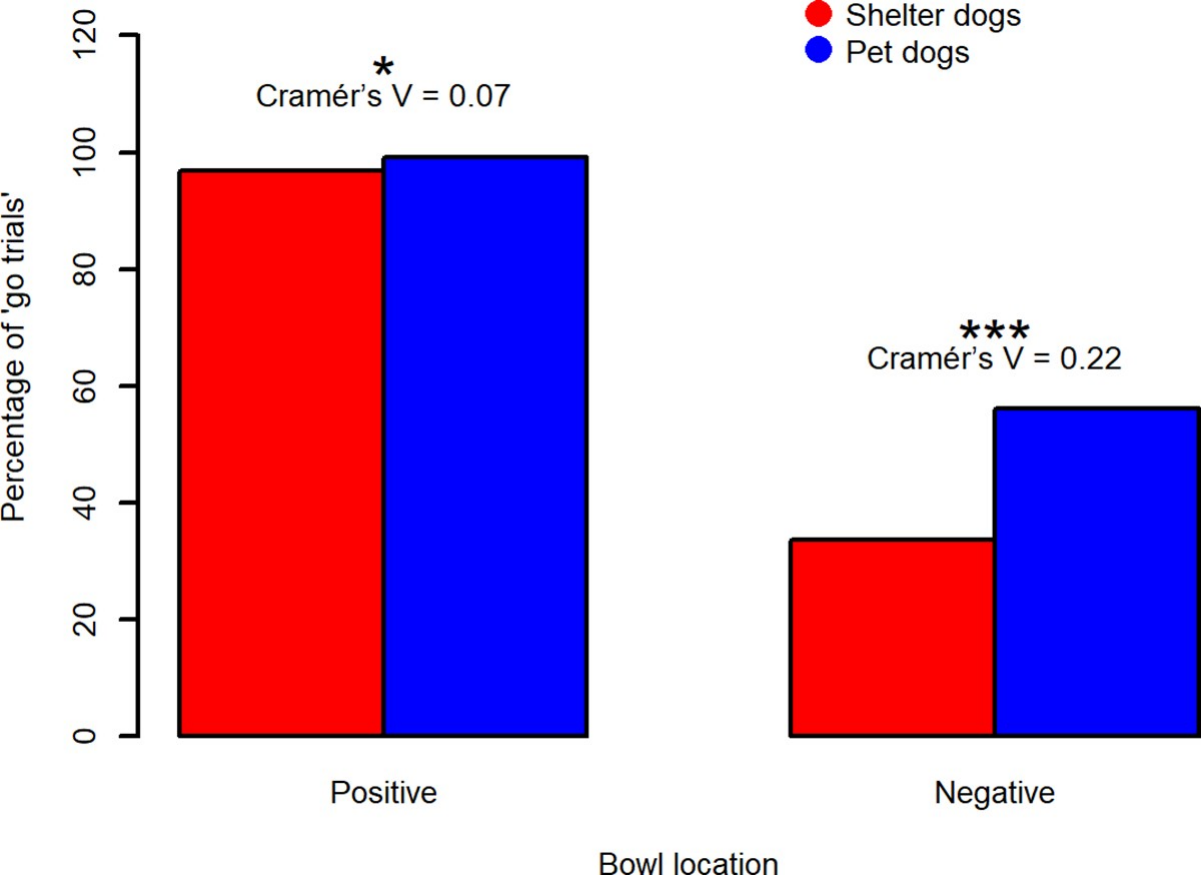
Percentage of “go trials” for the trained locations. A “go trial” is a trial in which the tested dog reached the bowl in less than 30 s. The percentage of “go trials” was calculated on the total number of trials of each type. Data are shown in percentage terms to take into account that the sheltered dog sample (N = 35) was larger than the pet dog sample (N = 31) and therefore the total number of trials was higher in the sheltered than in the pet dog sample. Significance codes: 0 ‘***’ 0.001, 0.01 ‘*’ 0.05.

Pearson's Chi-squared test with Yates' continuity correction revealed a significant association between occurrences of “go/no go trials” and the population of animals (sheltered dogs vs. pet dogs), for both P and N locations: (**P location:** X-squared = 6.3418, df = 1, p-value = 0.01; **N location:** X-squared = 52.99, df = 1, p-value < 0.0001). Standardised residuals showed that pet dogs exhibited more "go” responses than sheltered dogs, for both trained locations. However, Cramér’s V effect size coefficients revealed that, although the result was statistically significant for both P and N location, the association between the dogs’ behaviour (“go/no go” trials occurrences) and the population was weak regarding P location (Cramér’s V effect size = 0.07) and that the association was instead moderate regarding N location (Cramér’s V effect size = 0.22). Results are shown in Fig 7.

#### Optimism/Pessimism adjusted scores

**Fig 8:**
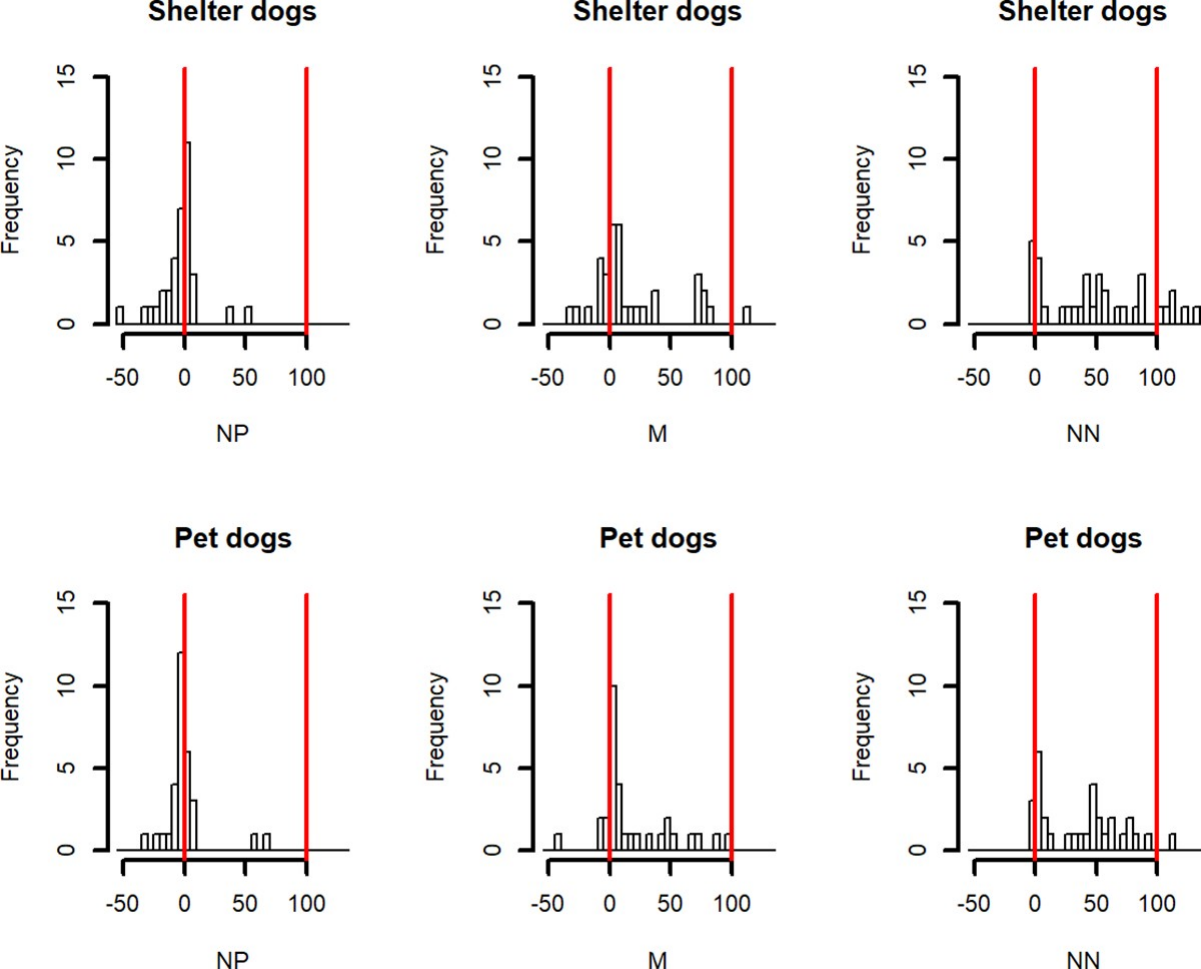
Optimism/pessimism adjusted score. Adjusted score’s frequency distribution for the three ambiguous locations: Near Positive (NP), Middle (M) and Near Negative (NN). Red vertical lines help finding 0 and 100 values.

The adjusted scores’ frequency distributions are graphically illustrated in Fig 8. Overall, as expected, dogs appeared to have a more optimistic attitude (lower adjusted scores) the closer to the positive position the bowl was located. The Linear Mixed-Effects model showed that the bowl location had a significant influence on the optimism/pessimism scores (df = 130, F = 37.11543, P-value < .0001). Post-hoc contrasts analysis revealed that NP adjusted scores were significantly lower than M adjusted scores (estimate = -20.438, Adjusted P-value, Bonferroni method = 0.0002) and that M adjusted scores were significantly lower than NN adjusted scores (estimate = -25.140, Adjusted P-value, Bonferroni method < 0.0001). No significant differences in the adjusted scores were found between sheltered and pet dogs.

It is worth noting that some adjusted scores values were lower than 0, i.e., the mean latency to reach the ambiguous location was lower than the mean latency to the reach the trained positive location and that some adjusted scores values were higher than 100, i.e., the mean latency to reach the ambiguous location was higher than the mean latency the reach the trained negative location.

## Discussion

The judgment bias test (JBT) represents a promising tool to assess the optimistic/pessimistic attitude of animals, a cognitive measure linked to emotional state and welfare. However, studies on dogs have not clearly established the link between emotions and judgment biases, with some studies yielding contradicting results [30, 32–34]. The reasons underlying these controversial results are not easy to determine given that the experimental paradigm and the statistical analysis of the data have been modified several times (see for a review [6, 7]).

The aim of the present paper was to evaluate the original paradigm of the judgment bias test on dogs [27] using an extensive statistical investigation (e.g., analysis of the training phase, responses to the trained locations during the testing phase, behaviour towards adjacent locations, variability in latencies to reach the bowl). To our knowledge, these issues have never been considered altogether. To make sure that our results were not biased by testing sheltered dogs (a population of animals under stress), all the analyses were mirrored on a population of pet dogs previously tested with an identical JBT paradigm by the same research group [26]. Where appropriate, a comparison between these two populations was also investigated.

Given that our dog populations were heterogeneous, we firstly verified the absence of potential influences of dogs’ characteristics (i.e., age, sex, reproductive status and, for sheltered dogs only, shelter location and length of stay in the shelter) on learning (i.e., minimum number of training trials required to reach the learning criterion) and on latencies to reach the bowls during the testing phase. No significant differences related to these variables emerged and learning was not influenced by the location of the reinforced bowl (left/right). Furthermore, results suggested that sheltered and pet dogs did not differ in learning skills in JBT (no differences in minimum number of training trials).

In line with previous studies [26–28, 30], results confirmed that dogs’ decision-making processes during the testing phase relied on the bowl position and not on the smell of treats.

The first critical point, detected through a proper model for repeated measures, regarded the dogs’ behaviour towards the bowl placed in adjacent locations. Latencies to reach the P and NP locations did not differ significantly in either population of animals (which is consistent with previous studies on dogs, [27, 30, 35]). Contrary to what one might have expected, latencies to reach the NP-M and M-NN locations also did not differ significantly, at least in the pet dog sample. This may reflect an optimistic judgment bias in pet dogs, but it also suggests that these dogs may have struggled to discriminate between adjacent locations and that some adjustments to the experimental design are required to facilitate discrimination (perhaps by increasing the distance between the locations). Some studies, according to dimensional models of affect, propose that the JBT is a useful tool to discriminate not only emotions with different valences, but also emotions with the same valence, but with different levels of arousal (see for a review [3]). For example, depression is characterized by a lower expectation of a positive event, potentially resulting in pessimistic responses to ambiguous stimuli that are similar to the trained positive cue [18, 52]; anxiety is instead characterized by a higher expectation of a negative event, resulting in reduced detection-latencies for cues appearing near the trained negative cue [53, 54]. To obtain this level of detail in the evaluation of emotions experienced by the individual, it is fundamental that dogs are able to discriminate properly among all the cues used during the test.

A second critical point regarded the median latency to reach the M location, expected to be the most ambiguous one. As can be seen in Fig 3, this latency was extremely short in both populations. Although this could be ascribed to a strong positive judgment bias, it must be considered that dogs could have reached this location so quickly because they were interested in the researcher who stood behind the bowl placed in M, rather than in the bowl itself. Previous studies highlighted this methodological caveat and provided evidence that the presence of a researcher can influence the dogs’ responses in the JBT. For instance, in Muller and colleagues’ study [33] the researcher did not stand behind M (the paradigm was slightly different from ours because dogs were able to see the researcher placing the bowl on the ground); these authors reported that the direction from which the researcher came when placing the bowl in M influenced the animals’ latencies (i.e., the latencies to reach M were shorter if the researcher approached the M position from the side of the positive location compared to the latencies measured when the researcher approached it from the side of the negative location). More recently, Kis and colleagues [39] compared dogs’ responses in spatial JBT when the researcher who placed the bowl on the ground was standing behind the M location (i.e., visible to the animal) and when the researcher was out of sight (i.e., invisible to the animal): they found that dogs reached the bowl faster if the researcher were visible. The strong bond between dogs and humans means that the presence of a person might have a confounding influence on test outcomes, especially towards dogs who experience infrequent human contact, and that they might get considerably aroused by human interaction [6]. Sheltered dogs often suffer from poor welfare due to social isolation [55–57], which could increase their motivation towards human social contacts.

Another element that was thoroughly analysed was the variability in dogs’ latencies to reach the bowl. Specifically, we explored for evidence of a learning effect between trials on the ambiguous locations (i.e., dogs after the first or the second trial may have learnt that the bowl placed in the ambiguous locations was empty). Analyses revealed no significant difference between trials in the animals’ latencies to reach the NP location, higher latencies to reach M in the second trial than in the first one and higher latencies to reach NN in the third trial compared to the second one. If a learning effect existed, then one would expect a similar pattern of response to all three ambiguous locations; the results from this study therefore suggest that there was not a proper learning effect between trials. Furthermore, it appears unlikely that dogs had learnt the association between the location of the bowl and its outcome in only one or two trials, given that the training phase required an average of 20 and 24 trials for sheltered and pet dogs respectively to learn this association. The differences in latencies between trials 1–2 (for the M location) and 2–3 (for the NN location) suggested instead a potential different optimistic/pessimistic attitude; for example more optimistic subjects may be inclined to keep reaching the bowl quickly, even after they did not find food in the first or second trial. On the basis of this result, we think that it is important to evaluate dogs’ latencies to approach each ambiguous location for all trials, rather than focusing solely on the first trial.

Regarding the trained locations, the Linear Mixed-Effect model confirmed an expected result: both sheltered and pet dogs behaved differently between P and N locations, supporting the hypothesis that dogs perceive these cues in a different light. However, it is worth noting that the variability in latencies was very high (see Fig 3); surprisingly, for both sheltered and pet dogs, the intra-dog variance (within-group error variance) was higher than inter-dog variance (between-group error variance) for both trained locations (see Fig 6). Whilst the inter-individual variability in latencies could be ascribed to differences in dogs’ personality, motivation or physical characteristics (such as the running speed), a high intra-individual variability entails inconsistency in behavioural responses by the same dog to the same stimulus. It is also worth taking into account that the nature of our data (censored at 30 seconds) may have caused a large intra-individual variance in latencies if only a few responses were different from the others (i.e., few ‘no go’ responses on P location or few ‘go’ responses on N location). A sporadic “no go” response towards P could be due to distraction, confusion or lack of interest [58]; a sporadic “go” response towards N could be due to the low risk involved in actually finding something ‘unpleasant’. However, intra-individual variability in latencies to reach N was extremely high. It should be pointed out that the JBT applied in the current study lasted nearly one hour per dog and this relatively long length of time (42 trials overall only in the testing phase) may have contributed to the observed inconsistency in behavioural responses, with some dogs maybe becoming tired or losing interest in the task.

Learning entails relatively permanent changes in behaviour and stable responses to the stimulus [59, 60]. In JBT, the consistency in latencies to reach the trained stimuli assures us that the dogs have indeed learned the association between the trained locations/outcomes and have reached a stable level of discrimination between P and N [61]. Our results suggest that, even if dogs behaved differently towards the two trained locations (mixed-effect models, see results), they might not have completely learned the association between the bowl location and the presence/absence of food, in particular with regards to the N location. By comparing the behaviour of the two populations during the testing phase, it emerged that pet dogs reached the bowl in N on more occasions and faster than sheltered dogs (see Figs 2 and 7); it might be hypothesized that in pet dogs, the association between the absence of food and the N location was weaker than in sheltered dogs. Rather than being a trained location, N could therefore have represented an ambiguous cue and pet dogs might be more optimistic subjects, taking a chance on the bowl placed in N (they also seemed more optimistic than sheltered dogs towards the NN location, see Fig 2).

The high intra-dog variability in latencies to reach N might also be due to the two stimuli (P and N) having a very different payoff. The baited bowl in P was a strong positive reinforcement, whereas the empty bowl in N could be considered to have held a mild negative salience. There was a low cost involved in a 4-metre walk to check the contents of the bowl placed in N and, in addition, it could be hypothesized that dogs had a strong prior conditioning that caused them to expect food in a bowl placed on the ground. As stated by Mendl and colleagues [3], when the “negative” outcome is mild (e.g., absence of an attractive stimulus as opposed to the presence of an aversive stimulus), subjects might take a chance on every location. As a matter of fact, dogs in the current study tended to reach the bowl even when in N (the percentage of ‘go trials’ was 34% for shelter dogs, 56% for pet dogs), and in these “go trials” dogs reached the bowl quickly (latencies were on average 9.6 and 6.78 s, for shelter and pet dogs respectively), although not as quickly as in P trials. This suggests that dogs behaved differently towards the two trained locations, probably due to a gradual extinction of the “go” response from the P to the N location.

We suggest that the difference between the payoff of the two stimuli was insufficient, therefore learning the reinforced/unreinforced nature of the trained stimuli could be difficult [62]. Given this methodological caveat, the assessment of pessimistic/optimistic attitude in JBT becomes arduous, since responses to ambiguous and trained cues are compared in order to detect a pessimistic/optimistic expectation of the negative/positive outcome previously experienced and learned. Therefore, in future studies it is important to make sure that subjects’ responses to trained stimuli are stable and well defined. To make this learning experience more reliable, one option may be to provide a negative cue that is more aversive or, perhaps, provide longer training sessions with a stronger learning criterion; a difference of 0.5 seconds between the slowest approach to P and the fastest approach to N in the last six training trials may be too slight. The learning criterion could entail a higher and consistent difference between the slowest approach to P and the fastest approach to N, for more than 6 training trials. However, this way the duration of the training phase would inevitably be extended, with the disadvantage of lengthening the duration of the test even more.

Another concern that can be raised when analysing data from the training phase, is whether this paradigm is truly suitable for dogs. During training, 69% of sheltered dogs and 78% of pet dogs fulfilled the learning criterion and were able to move to the testing phase. Müller and colleagues [33], using the same paradigm, reported a similar percentage (75%) of dogs who achieved criterion. Even if these figures still represent the majority of dogs, an inclusion bias could be hypothesised; that is, dogs who struggle to reach the criterion and are therefore excluded from the test, may be the ones experiencing a negative, or vice versa, a positive mood. As an example, Willen and colleagues [31] found that fearful dogs needed more training trials than non-fearful ones to reach a similar learning criterion. We suggest that the paradigm may not be suited for dogs who, for whatever reason, struggle to learn the association between locations and outcomes. A stronger difference between the payoff of trained stimuli could shorten and simplify the learning process and strengthen the association between trained locations and their outcomes.

Despite the highlighted methodological caveats, sheltered dogs appeared to be more pessimistic than pet dogs when comparing the raw latency to reach the five locations using a proper mixed-effects model (see Fig 2). In order to compare our results with previous studies, an “adjusted score” of optimism/pessimism was calculated in line with Mendl and colleagues [27]. Overall, as expected, dogs appeared to have a more optimistic attitude (lower adjusted scores) the closer to the positive position the bowl was located. However, some patterns of response deserved a critical analysis. First of all, this approach implies that latencies are averaged across several trials of the same type and therefore this approach reduces variability in the data (which, as we discussed above, should not be ignored) and does not take into account the dependencies in the repeated measures. Secondly, some scores were lower than 0, indicating that dogs reached the ambiguous locations faster than the positive one, whereas some scores were higher than 100, indicating that dogs reached the ambiguous locations slower than the negative one. In addition, considering the high intra-individual variability in latencies to reach P and N (used as anchor in the formula to calculate adjusted scores), we recommend that the interpretation of these adjusted scores as measures of optimism/pessimism should be cautious. Furthermore, statistical analysis applied on the pessimism/optimism adjusted score did not detect a more pessimistic attitude in shelter dogs towards the NN location compared to pet dogs. Therefore, the statistical approach that analyses raw latencies to reach the bowl in each trial seems to be preferable.

## Conclusions

The aim of this study was to consider a spatial JBT widely applied on dogs (e.g. [30, 32–34]) to investigate methodological and statistical criticalities highlighted by previous reviews [7, 42, 58] and to propose possible solutions and improvements. Our analyses revealed that there are some caveats in the judgment bias tests methodology. Firstly, dogs who passed the training did not behave differently towards bowls placed in adjacent locations; secondly, a great inconsistency in response to the trained stimuli was found, perhaps because dogs did not fully learn the association between the bowl location and the presence/absence of food. Finally, dogs’ responses may have been influenced by the presence of the researcher behind the M position.

These outcomes point to a necessity of methodological adjustments to improve the experimental set-up: a bigger difference between payoff of the trained locations could help subjects to learn the association between the bowl location and its outcome, may allow more individuals to pass the training and thus take part in the test [7].

All of this is essential to properly detect optimistic and pessimistic responses to ambiguous cues and therefore to infer the emotional state of subjects. Despite the highlighted methodological caveats, by analysing the dogs’ behaviour using raw latencies and a proper mixed-effects model, sheltered dogs appeared to be more pessimistic than pet dogs. However, the significant difference between the two populations, even towards the N location, raises some doubts about the reliability of this JBT paradigm. In this paper, we suggest some improvements that could be implemented in an effort to establish a valid cognitive bias tool for dogs. Such an approach could be particularly valuable for sheltered animals, allowing individuals at risk of reduced emotional welfare to be identified and targeted for interventions aimed at improving quality of life.

## Supporting information

S1 Appendix (DOCX)
