# Supplementary material for: Using judgment bias test in pet and shelter dogs (Canis familiaris): Methodological and statistical caveats
Source: PLoS One. 2020 Oct 27;15(10):e0241344. doi: 10.1371/journal.pone.0241344 (PMC7591058; doi:10.1371/journal.pone.0241344)
Supplement: S1 Appendix — (DOCX) [file pone.0241344.s001.docx]

**Table A. Characteristics of shelter dogs tested with judgment bias test**

| Name | Breed | Age (years) | Sex | Reproductive status | Shelter | Permanence (days) |
| --- | --- | --- | --- | --- | --- | --- |
| Abbey | Mixed breed | 4 | F | Neutered | BARK | 30 |
| Athos | German Shepherd | 10 | M | Entire | ANPANA | 180 |
| Blackjack | Mixed breed | 2 | M | Neutered | BARK | 7 |
| Bobby | Lhasa Apso | 2 | M | Neutered | BARK | 16 |
| Chinotto | Mixed breed | 1 | M | Entire | ANPANA | 10 |
| Dodger | Mixed breed | 1,5 | M | Neutered | BARK | 21 |
| Enjoy | Mixed breed | 2 | M | Entire | ANPANA | 23 |
| Eric | Mixed breed | 9 | M | Entire | ANPANA | 180 |
| Fudge | Lhasa Apso | 8 | M | Entire | BARK | 29 |
| Honeycomb | Mixed breed | 2 | F | Neutered | BARK | 7 |
| Jack | Mixed breed | 3 | M | Entire | ANPANA | 38 |
| Lea | Mixed breed | 2 | F | Entire | ANPANA | 55 |
| Lucky_shelter | Mixed breed | 10 | F | Neutered | BARK | 227 |
| Lyca | Mixed breed | 1 | F | Entire | ANPANA | 60 |
| Max_shelter | Mixed breed | 1 | M | Entire | ANPANA | 60 |
| Miriam | Mixed breed | 4 | F | Entire | ANPANA | 365 |
| Monty | Border Collie | 6 | M | Neutered | BARK | 14 |
| Pepe | Mixed breed | 2 | M | Neutered | ANPANA | 150 |
| Pepper | Mixed breed | 1 | F | Neutered | BARK | 17 |
| Pippin | Mixed breed | 5 | F | Neutered | BARK | 10 |
| Reilly | English Springer Spaniel | 1 | M | Neutered | BARK | 12 |
| Ringo | Mixed breed | 2 | M | Entire | ANPANA | 150 |
| Rocco | Mixed breed | 5 | M | Entire | ANPANA | 1095 |
| Rocky | Mixed breed | 10 | M | Entire | ANPANA | 1460 |
| Roxy | Lhasa Apso | 8 | F | Neutered | BARK | 28 |
| Rufus | Griffon Bruxelloise | 1 | M | Neutered | BARK | 25 |
| Rum | Mixed breed | 3 | M | Neutered | BARK | 15 |
| Ryder | German Shepherd | 9 | M | Entire | ANPANA | 60 |
| Scatoletta | Mixed breed | 6 | M | Entire | ANPANA | 116 |
| Shame | Mixed breed | 3 | M | Entire | BARK | 7 |
| Socks | Mixed breed | 3 | M | Entire | ANPANA | 90 |
| Soda | English Springer Spaniel | 4 | F | Neutered | BARK | 44 |
| Sooty | Jack Russell Terrier | 4 | F | Neutered | BARK | 26 |
| Trixie | Labrador Retriever | 8 | F | Neutered | BARK | 14 |
| Tyson | Mixed breed | 3 | M | Neutered | ANPANA | 365 |

**Table B. Characteristics of pet dogs tested with judgment bias test**

| Name | Breed | Age (years) | Sex | Reproductive Status |
| --- | --- | --- | --- | --- |
| BebeP | Mixed Breed | 1,5 | F | Entire |
| Buddy | Mixed Breed | 8,0 | M | Neutered |
| Ellie | Mixed Breed | 4,5 | F | Neutered |
| Finn | Mixed Breed | 5,0 | M | Neutered |
| Finn_R | Schnauzer | 8,5 | M | Neutered |
| Fionn | Mixed Breed | 5,0 | M | Neutered |
| Gina | Mixed Breed | 4,5 | F | Neutered |
| Loki | Mixed Breed | 2,0 | M | Neutered |
| Lola | Mixed Breed | 5,0 | F | Neutered |
| LouieP | Miniature Schnauzer | 3,0 | M | Neutered |
| Lucky | Jack Russell Terrier | 3,0 | F | Neutered |
| Luna | Mixed Breed | 10,0 | F | Neutered |
| Maisie | Dachshund | 6,0 | F | Neutered |
| Max | Mixed Breed | 7,0 | M | Neutered |
| Max_C | Labrador Retriever | 2,5 | M | Entire |
| Mia | Mixed Breed | 6,5 | F | Neutered |
| Penny | Labrador Retriever | 3,0 | F | Neutered |
| Poppy | Staffordshire Bull Terrier | 3,5 | F | Neutered |
| PoppyP | Mixed Breed | 1,0 | F | Entire |
| Riggins | Mixed Breed | 2,5 | M | Neutered |
| Romeo | Mixed Breed | 4,0 | M | Neutered |
| Ruby | Labrador Retriever | 6,0 | F | Neutered |
| Ruby_L | Labrador Retriever | 7,0 | F | Entire |
| Ruby_V | Hungarian Short-haired Pointer (Vizla) | 6,0 | F | Entire |
| Rupert | Mixed Breed | 1,0 | M | Neutered |
| Sophie | Dachshund | 9,0 | F | Neutered |
| Suzie | Mixed Breed | 1,0 | F | Neutered |
| Tara | Irish Red Setter | 2,0 | F | Neutered |
| TobyP | Yorkshire Terrier | 8,0 | M | Neutered |
| Wizard | Mixed Breed | 3,0 | M | Neutered |
| Zara | Mixed Breed | 2,0 | F | Neutered |
